# Serum proteomic and metabolomic profiling of hepatocellular carcinoma patients co-infected with *Clonorchis sinensis*

**DOI:** 10.3389/fimmu.2024.1489077

**Published:** 2025-01-07

**Authors:** Zeli Tang, Caibiao Wei, Xueling Deng, Qiumei Lin, Qiping Hu, Shitao Li, Jilong Wang, Yuhong Wu, Dengyu Liu, Min Fang, Tingzheng Zhan

**Affiliations:** ^1^ Department of Cell Biology and Genetics, School of Basic Medical Sciences, Guangxi Medical University, Nanning, China; ^2^ Key Laboratory of Longevity and Aging-related Diseases of Chinese Ministry of Education, Guangxi Medical University, Nanning, China; ^3^ Key Laboratory of Basic Research on Regional Diseases, Education Department of Guangxi Zhuang Autonomous Region, Guangxi Medical University, Nanning, China; ^4^ Department of Clinical Laboratory, Guangxi Medical University Cancer Hospital, Nanning, China; ^5^ Department of Parasitology, School of Basic Medical Sciences, Guangxi Medical University, Nanning, China; ^6^ Engineering Research Center for Tissue & Organ Injury and Repair Medicine, Guangxi Medical University Cancer Hospital, Nanning, China

**Keywords:** *Clonorchis sinensis*, hepatocellular carcinoma, prognosis, proteomic, metabolomic

## Abstract

**Background:**

*Clonorchis sinensis* (*C. sinensis*) infection is a significant risk factor for hepatocellular carcinoma (HCC), yet its underlying mechanisms remain poorly understood. This study aimed to investigate the impact of *C. sinensis* infection on the serum proteomic and metabolomic profiling of HCC patients, focusing on the potential mechanisms.

**Method:**

A retrospective clinical analysis was conducted on 1121 HCC patients, comparing those with and without *C. sinensis* infection. The influence of *C. sinensis* on serum proteome and metabolome in HCC was further assessed.

**Result:**

*C. sinensis* infection correlated with a younger age at cancer onset, male predominance, advanced cancer stage, liver cirrhosis, and microvascular invasion in HCC patients. It also associated with shorter overall survival (OS) and recurrence-free survival (RFS). The levels of blood lipids (e.g., APO-A, HDL-C, and TG) were significantly altered after *C. sinensis* infection. Proteomic and metabolomic analyses revealed metabolic reprogramming caused by *C. sinensis*, with excessive depletion of argininosuccinate synthase (ASS) and D-glucose as potential factors in *C. sinensis*-associated HCC malignancy. Key molecules ILF2, CNN2, OLFM4, NOTCH3, and LysoPA were implicated in HCC progression. Furthermore, *C. sinensis* triggered inflammation, insulin resistance, and pro-tumor immune escape, and exacerbated the complication of degenerative diseases.

**Conclusion:**

This study not only provides compelling evidence for elucidating the mechanisms underlying *C. sinensis*-mediated HCC development but also identifies potential therapeutic targets for HCC patients co-infected with *C. sinensis*.

## 1 Introduction

Hepatocellular carcinoma (HCC) is the most common histological type of primary liver cancer, accounting for 70%-85% of cases (1, 2). HCC is a leading cause of cancer-related mortality worldwide and poses a significant challenge to global healthcare. The overall 5-year survival rate for HCC is less than 30% (1, 3). The main risk factors for HCC include chronic hepatitis B virus (HBV) and hepatitis C virus (HCV) infections, excessive alcohol consumption, exposure to dietary toxins like aflatoxin and aristolochic acid, and metabolic liver diseases (4, 5). There is growing evidence suggesting that helminth infections may contribute to various types of human cancers. Specifically, *Clonorchis sinensis* (*C. sinensis*), a parasite that resides in the bile ducts, can cause progressive hepatitis and fibrosis, which is closely associated with the development of hepatobiliary malignancies (6, 7).

In 2009, the International Agency for Research on Cancer classified *C. sinensis* as a Group 1 biological carcinogen for humans (8). Recent research has further supported the role of *C. sinensis* in the development of HCC and its adverse impact on prognosis. A rat model study has shown that *C. sinensis* increases the risk of HCC by stimulating proliferation of hepatic progenitor cells (9). Clinical retrospective analyses demonstrated that *C. sinensis* is a poor prognostic factor for HCC following hepatectomy, regardless of co-infection with HBV (10, 11). Additionally, *C. sinensis* has been found to up-regulate CK19 and EpCAM, which are stem cell markers for cancer, thereby promoting the malignant progression of HCC (11). Furthermore, *in vitro* cell experiments have confirmed that proteins of granulin, GIIIsPLA2, and severin of *C. sinensis* contribute to the malignant development of HCC cell lines (12–14).

The liver, as a highly metabolic organ, plays pivotal roles in digestion, detoxification, secretion, and storage. Metabolic dysregulation serves as a hallmark of HCC development (15, 16). Prior studies conducted on mice and rats have shown that infection with *C. sinensis* significantly modifies both the proteomic and metabolic profiles in the liver and serum (17, 18). Nonetheless, it remains unclear whether *C. sinensis* instigates gene expression and metabolic reprogramming in HCC patients, consequently contributing to the progression of HCC. Hence, this study embarked on a systematic analysis to evaluate the impact of *C. sinensis* on clinical parameters in HCC patients, with a specific emphasis on changes in serum biochemical indexes. Furthermore, a combination of metabolomics and proteomics techniques was employed to elucidate the effects of *C. sinensis* on the serum metabolome and proteome in HCC, thereby shedding light on the pathogenic mechanisms underlying *C. sinensis*-related HCC and identifying potential therapeutic targets.

## 2 Methods

### 2.1 Ethics statement

The retrospective analysis in this study was conducted at Guangxi Medical University Cancer Hospital, between January 2014 to December 2022. Serum samples for proteomics analysis and metabolomics analysis were collected at Guangxi Medical University Cancer Hospital between September 2022 and February 2023. This study adhered to the ethics outlined in the Declaration of Helsinki and received approval from the Ethics Committee of Guangxi Medical University Cancer Hospital.

### 2.2 Study population and data collection

A total of 2462 patients diagnosed with HCC underwent curative resection. The inclusion criteria for retrospective study were as follows: (1) HCC confirmed by postoperative pathological analysis, (2) no history of previous anti-cancer therapy, (3) absence of concurrent malignant tumors, (4) availability of comprehensive laboratory, and pathological. On the other hand, the exclusion criteria consisted of the following: (1) individuals with a history of anti-tumor therapy such as radiotherapy, or chemotherapy (n=205), (2) cases without a clear pathological diagnosis (n=106), (3) patients with other tumor diseases (n=221), (4) recurrence of HCC (n=196), (5) unavailability of comprehensive laboratory and pathological data (n=613).

The diagnostic criteria for clonorchiasis were as follows, with any one of the subsequent conditions deemed sufficient for establishing a diagnosis (19). (1) Intraoperative or postoperative pathological examination revealing the presence of adult *C. sinensis* in the liver or gallbladder. (2) Preoperative fecal examination showing the presence of *C. sinensis* eggs. Based on above criteria, a total of 1121 patients were included in this study, among whom 118 exhibited concomitant *C. sinensis* infection along with HCC. Therefore, the patients were divided into two groups: the HCC group (without *C. sinensis* infection) and the HCC_*Cs* group (HCC combined with *C. sinensis* infection).

The data collection process encompassed multiple aspects, including: (1) General information: gender and age. (2) Hematological tests: including various tumor markers such as alpha-fetoprotein (AFP), carcinoembryonic antigen (CEA), and hepatitis B surface antigen (HBsAg). (3) Pathological indicators: liver cirrhosis, number of tumors, tumor differentiation degree (based on the Edmondson-Steiner histological grading system) and the presence of microvascular invasion (MVI). After excluding those without follow-up data (n=216), a total of 905 patients with HCC were included in the calculation of overall survival (OS) and recurrence-free survival (RFS).

**Figure 1:**
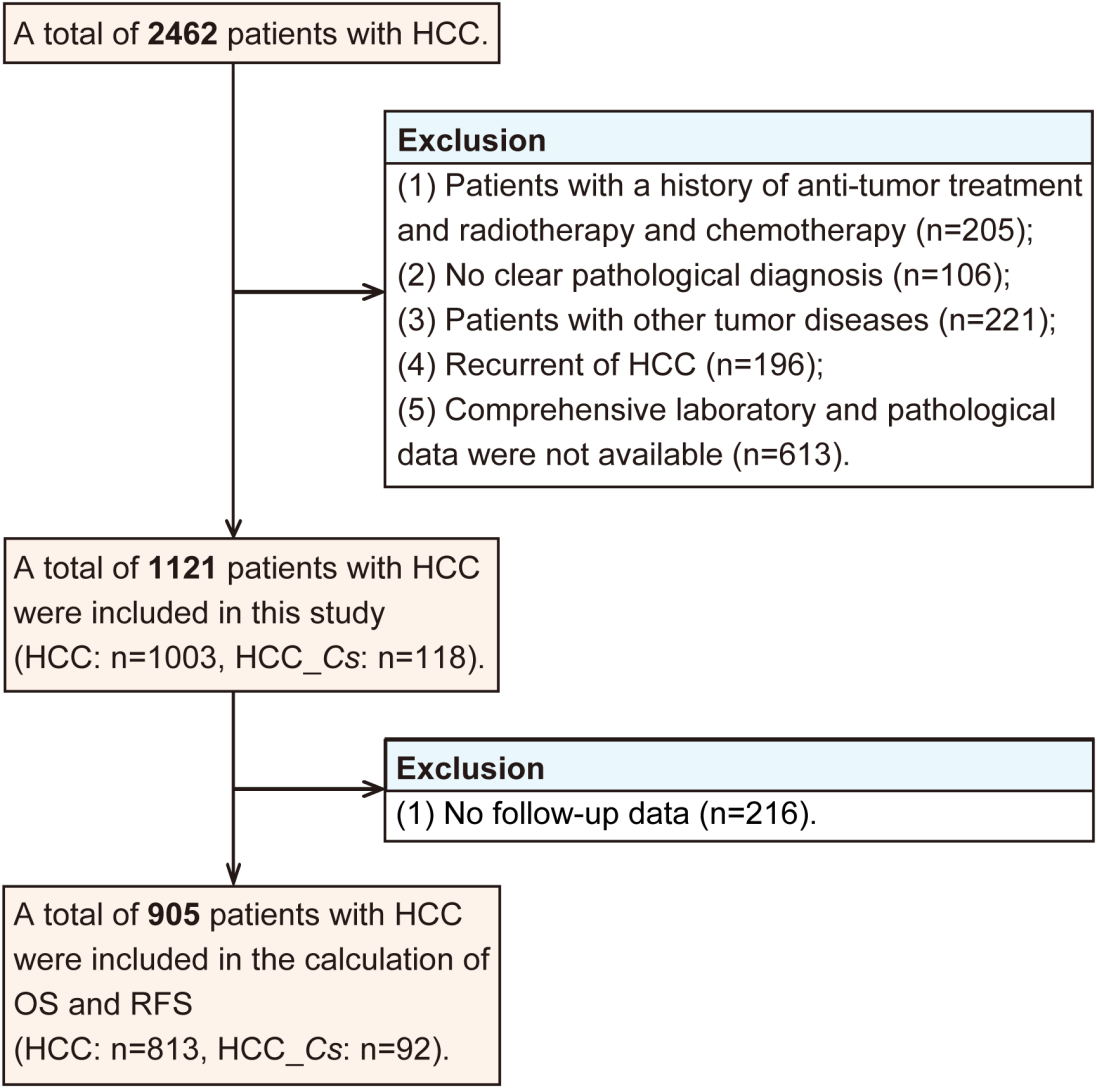
The flow of study participant.

Excluding those with no follow-up data (n=216), a total of 905 patients with HCC were subjected to the calculation of OS and RFS. The exclusion process for participants in this study was graphically displayed in **Figure 1**.

### 2.3 Laboratory methods

The concentration of alanine aminotransferase (ALT), aspartate aminotransferase (AST), lipase (LPS), apolipoprotein A (APO-A), apolipoprotein B1 (APO-B1), high density lipoprotein cholesterol (HDL-C), triglyceride (TG), apolipoprotein A1/apolipoprotein B1 (A1/B1), transferrin (TRF), and blood ammonia (AMM) were determined utilizing a Siemens ADVIA 2400 chemistry analyzer (Siemens, Munich, Germany). Meanwhile, the levels of AFP and CEA were quantified employing an Abbott I2000SR analyzer (Abbott, Illinois, USA).

### 2.4 Follow-up routine

All patient follow-up information was diligently managed by professionals. Disease status or date of death was determined through telephone contact or an outpatient monitoring system. Tumor recurrence was identified by analyzing radiological observations from CT or MRI scans, with a focus on discerning characteristic enhancement patterns that indicate of intrahepatic recurrence. For extrahepatic tumors or those with atypical HCC imaging traits, verification was secured through biopsy. Patients underwent systematic monitoring at specific time intervals subsequent to their surgical procedures. OS was calculated by determining the time between the date of liver resection and the date of death or last follow-up until August 30, 2023. RFS was defined as the time interval between the date of liver resection and the date of death or last follow-up also until August 30, 2023.

### 2.5 Proteomic analyses

For proteomic analyses, 8 serum samples were selected from HCC and *C. sinensis* positive HCC (HCC_*Cs*) patients, respectively. All the serum samples were obtained from patients with HCC diagnosed on first admission, with no history of tumor treatment and no concomitant tumors. The proteins of both groups were detected using 4D Label Free Quantitative proteomics technology. EASY-nLC 1000 system (Thermo, Massachusetts, USA) and a timsTOF Pro2 mass spectrometer (Bruker, Karlsruhe, Germany) were used to solubilize peptides and subsequently analyzed by Untargeted liquid chromatography-tandem mass spectrometry (LC-MS/MS). The MS/MS scan range was set from 100 to 1700 m/z. Data acquisition was performed using the parallel accumulation serial fragmentation (PASEF) acquisition mode. Raw data were searched using MaxQuant software. All data were analyzed through the online cloud platform of Majorbio (https://www.majorbio.com/). DEPs were classified based on three categories: biological process (BP), cellular component (CC), and molecular function (MF) using Gene Ontology (GO, http://geneontology.org/) annotation. Pathway enrichment analysis of DEPs was conducted using the Kyoto Encyclopedia of Gene and Genomes (KEGG) pathway database (http://www.genome.jp/kegg/). The STRING protein interaction database (https://string-db.org/) was used to analyze the protein-protein interaction (PPI) network.

### 2.6 Metabolomic analyses

12 serum samples were selected from the HCC and HCC_*Cs* patient groups, respectively. All the serum samples were obtained from patients with HCC diagnosed on first admission, with no history of tumor treatment and no concomitant tumors. LC-MS/MS was performed to detect metabolites in both groups. The subsequent analytical procedures were executed by Majorbio Bio-Pharmm Technology Co., Ltd. (Shanghai, China) according to the reference method described below (20): briefly, after the samples were processed accordingly, the tests were performed on a UHLC-Q Active HF-X system (Thermo, Massachusetts, USA). The samples were separated by HSS T3 column (Waters, Milford, USA) and then detected by mass spectrometry. Mass spectrometry signals were acquired in both positive and negative ion scanning modes. The parameters were set as follows: spray voltage of 3.5 kV and -3.5 kV, a scanning range of 70-1050 m/z, normalized collision energy set at 20-40-60V, and primary and secondary mass spectrometry resolutions of 60000 and 7500, respectively. Data was collected using the DDA mode. Subsequently, the raw metabolic data was processed using Progenesis QI (Waters Corporation, Milford, USA) with multiple analytical techniques to generate a comprehensive data matrix for further analysis, including relative standard deviation (RSD) distribution, Orthogonal Partial Least-Squares Discriminant Analysis (OPLS-DA) analysis, Venn diagram analysis, identification of differentially expressed metabolites (DEMs), screening of significant metabolites, cluster analysis of DEMs, and KEGG pathway analysis.

### 2.7 Correlation analyses of proteomics and metabolomics

Correlation analyses were conducted on the Majorbio cloud platform to examine the relationships between DEPs and DEMs based on the proteomics and metabolomics datasets. The analyses included two-way orthogonal partial least squares (O2PLS) analysis, functional enrichment analysis, Venn diagram analysis, and expression correlation analysis.

### 2.8 Statistical analysis

Statistical analysis was performed using IBM SPSS Statistics software version 26.0 and R version 4.2.1. Intergroup differences for categorical data, presented as ratios, were compared using either the Chi-square test or Fisher’s exact test. The Mann-Whitney U test was used to compare non-normal continuous data. Survival curves were generated using the Kaplan-Meier method, and intergroup comparisons of OS and RFS rates were performed using the log-rank test. For proteomic analyses, Student’s t-test (two-tailed) was used with a *P* value < 0.05 and |FC| ≥ 2 as the filtering criteria. For metabolomics data, FC analysis and T test/non-parametric test were used to analyze the differences between the two groups of samples. Pearson’s correlation coefficient and Fisher’s exact test were used for correlation analysis of DEPs and DEMs, as well as correlation analysis of proteomics and metabolomics pathway enrichment, respectively. All statistical tests were two-sided, and *P* < 0.05 was considered to be statistically significant.

## 3 Result

### 3.1 Population characteristics

A total of 1121 patients were included in the study, comprising 988 males and 133 females, with an average age of 51 ± 10 years old. Detailed clinical baseline data for each group were displayed in **Table 1**. Statistical analysis revealed significant differences in gender, age, BCLC stage, liver cirrhosis, MVI, AFP, and HBsAg between the HCC_*Cs* group and *C. sinensis* negative HCC group (*P* < 0.05, **Table 1**). The prevalence of males was significantly higher in the HCC_*Cs* group compared to the HCC group (97.5% vs. 87.0%, *P*=0.001). The BCLC stage was more advanced in the HCC_*Cs* group than the HCC group (BCLC_B~C 56.8% vs. 40.4%, *P*=0.001). Moreover, the HCC_*Cs* group exhibited higher rates of cirrhosis (66.1% vs. 53.8%) and MVI (58.5% vs. 46.3%) (all *P* < 0.05). The AFP (*P*=0.004) and HBsAg (*P*=0.018) values of patients in the HCC_*Cs* group were significantly higher and lower than those in the HCC group, respectively. However, no statistically significant differences were observed in tumor size, number of tumors, Edmondson grade, CEA, and HCV (**Table 1**).

**Table 1:** Patient demographics and clinical characteristics.

Characteristics	HCC	HCC_*Cs*	*P*
	No. (%)	No. (%)	
**Total**	1003	118	
**Gender**			
Male	873(87.0%)	115(97.5%)	0.001
Female	130(13.0%)	3(2.5%)	
**Age(years)**			0.049
<60	661(65.9%)	67(56.8%)	
≥60	342(34.1%)	51(43.2%)	
**BCLC stage**			0.001
A	598(59.6%)	51(43.2%)	
B~C	405(40.4%)	67(56.8%)	
**Tumor size**			0.303
<5cm	458(45.7%)	48(40.7%)	
≥5cm	545(54.3%)	70(59.3%)	
**Liver cirrhosis**			0.011
Negative	463(46.2%)	40(33.9%)	
Positive	540(53.8%)	78(66.1%)	
**Edmondosom grade**			0.592
I-II	494(49.6%)	54(47.0%)	
II-IV	502(50.4%)	61(53.0%)	
NA	7	3	
**Number of tumor**			0.586
<2	830(82.8%)	100(84.7%)	
≥2	173(17.2%)	18(15.3%)	
**MVI**			0.03
Negative	539(53.7%)	51(41.5%)	
Positive	464(46.3%)	67(58.5%)	
**CEA**			0.443
<5μg/L	883(88.0%)	101(85.6%)	
≥5μg/L	120(12.0%)	17(14.4%)	
**AFP**			
<400ng/mL	622(62.0%)	57(48.3%)	0.004
≥400ng/mL	381(38.0%)	61(51.7%)	
**HBsAg**			0.018
Negative	146(14.6%)	27(22.9%)	
Positive	857(85.4%)	91(77.1%)	
**Anti-HCV**			0.229
Negative	991(98.8%)	115(97.5%)	
Positive	12(1.2%)	3(2.5%)	

### 3.2 The association between *C. sinensis* infection and poor prognosis in HCC patients

**Figure 2:**
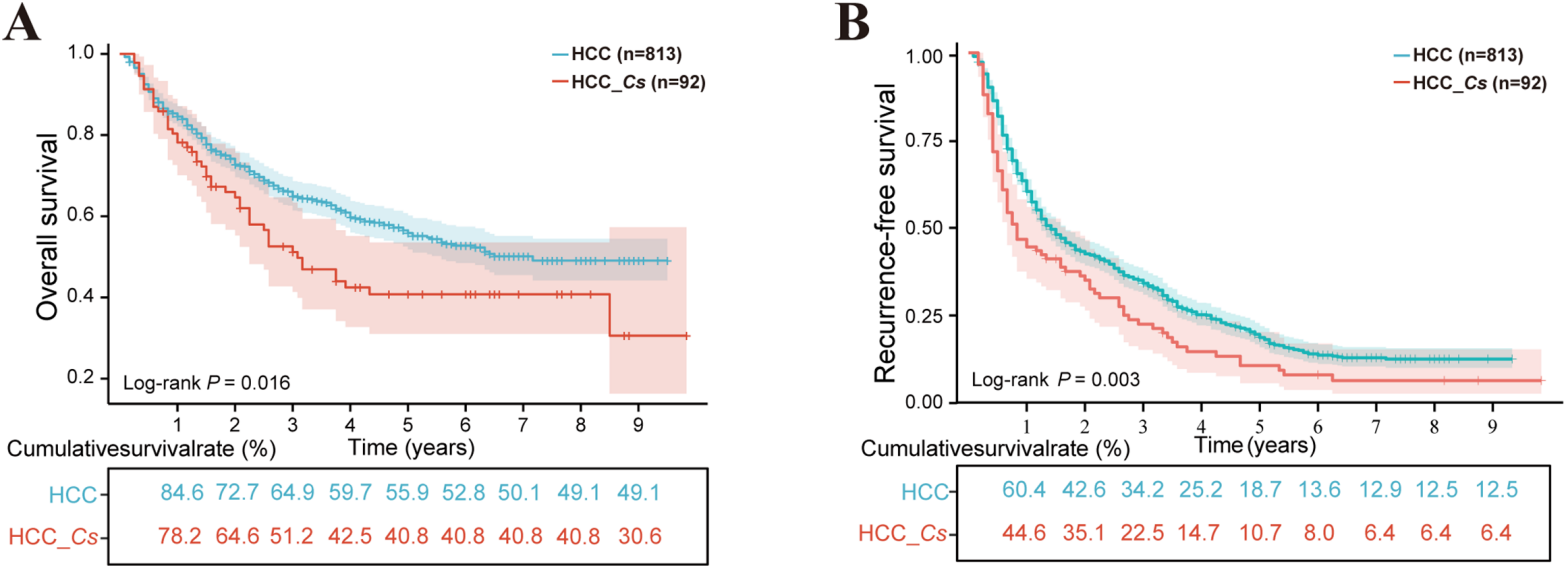
The influence of *C. sinensis* on the prognosis of patients with HCC after hepatectomy. **(A)** *C. sinensis* for overall survival. **(B)** *C. sinensis* for recurrence-free survival. *P* value by log-rank test.

A total of 813 patients with HCC and 92 patients with HCC_*Cs* were included in the study for the analysis of OS and RFS. The median follow-up time for OS was 77 months (range 1-118 months), while the median follow-up time for RFS was 16 months (range 1-118 months). At the last follow-up, there were 509 (62.6%) and 43 (46.7%) patients alive in the HCC group and HCC_*Cs* group, respectively. Moreover, among the patients who underwent RFS follow-up, 177 patients (21.7%) in the HCC group and 11 patients (11.9%) in the HCC_*Cs* group did not experience recurrence. The HCC_*Cs* patients had worse OS compared to the HCC patients, with three-year survival rates of 51.2% vs. 64.9% and five-year survival rates of 40.8% vs. 55.9% (*P*=0.016, **Figure 2A**). Similarly, compared with HCC patients, patients with HCC_*Cs* had lower RFS rates (three years: 34.2% vs. 22.5%; five years: 18.7% vs. 10.7%, *P*=0.003, **Figure 2B**).

### 3.3 Serum index characteristics of the study population

**Figure 3:**
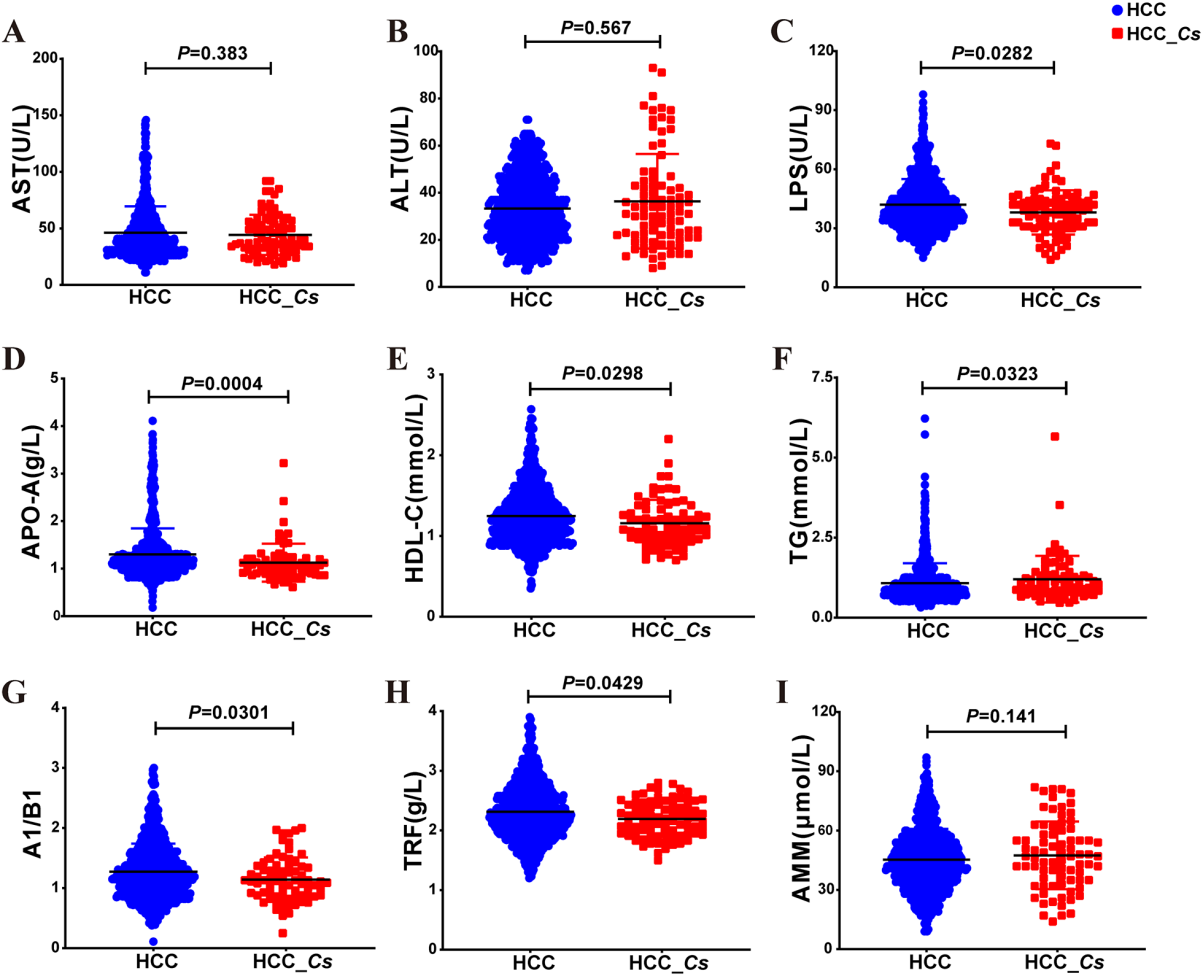
Serum indicator levels between HCC group and HCC_*Cs* group in large samples. Serum levels of AST **(A)**, ALT **(B)**, LPS **(C)**, APO-A **(D)**, HDL-C **(E)**, TG **(F)**, A1/B1 **(G)**, TRF **(H)**, and AMM **(I)** in large samples of HCC group and HCC_*Cs* group patients were detected. *P* value by Mann-Whitney U test.

Serum biochemical analysis revealed that the levels of LPS, APO-A, HDL-C, A1/B1, and TRF were significantly lower in the HCC_*Cs* group compared to the HCC group (*P* < 0.05, **Figures 3C-E, G, H**). Conversely, the levels of triglyceride (TG) were significantly higher (*P* < 0.05, **Figure 3F**). Although there was no significant difference in the levels of aspartate aminotransferase (AST), alanine aminotransferase (ALT), and blood ammonia (AMM) between the two groups, the levels of ALT and AMM were increased in the HCC_*Cs* group (*P* > 0.05, **Figures 3A, B, I**). Furthermore, the serum samples (12 cases in each group) subsequently used for proteomic and metabolomic analyses also exhibited similar trends in these indicators (**Supplementary Figure S1**).

### 3.4 Annotation and functional enrichment of differentially expressed proteins (DEPs) in the serum of HCC and HCC_*Cs* patients

**Figure 4:**
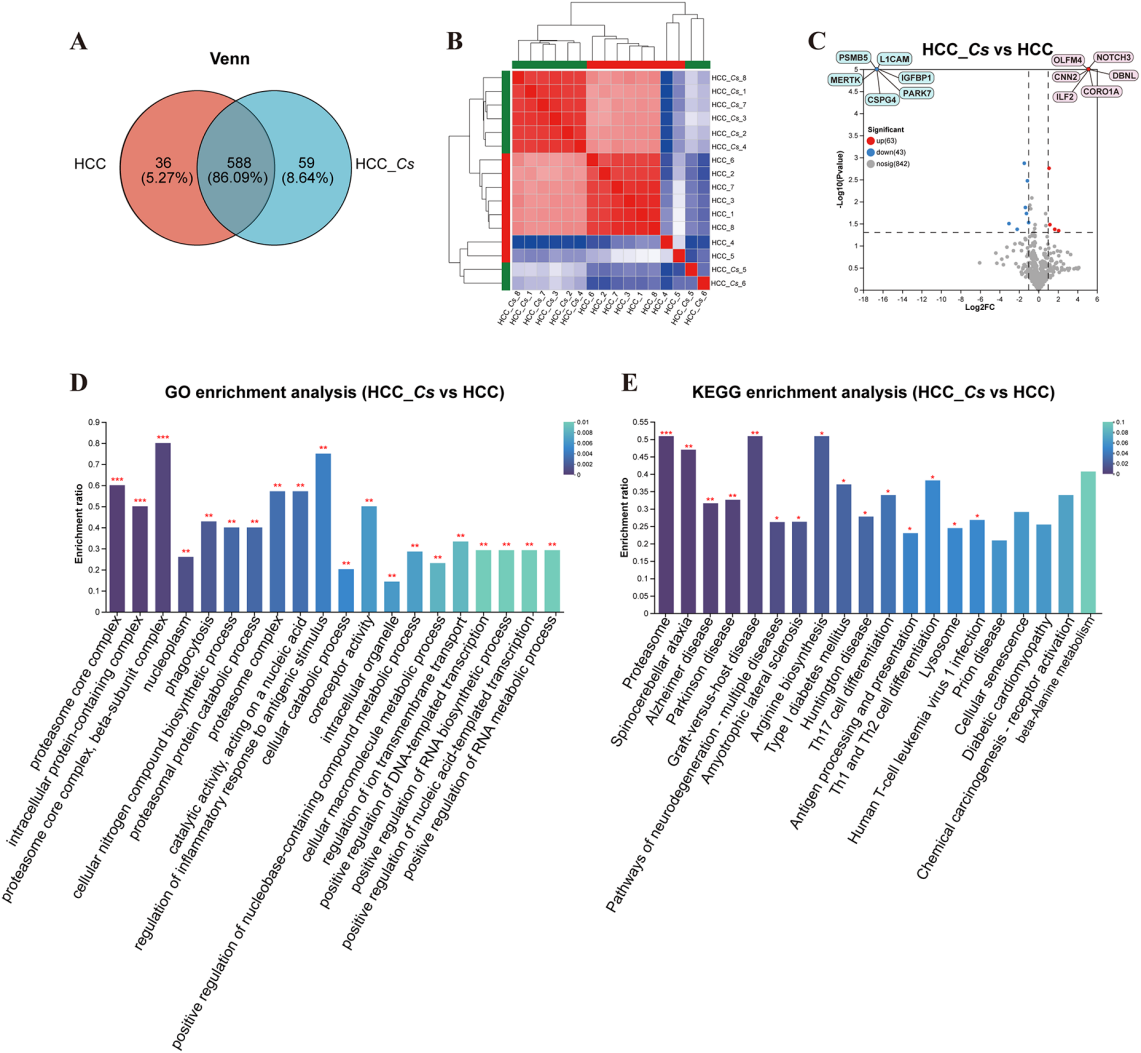
Identification and enrichment analysis of serum DEPs between HCC group and HCC_*Cs* group. **(A)** Venn diagram of serum proteins identified between two groups. **(B)** Correlation heatmap of serum samples. **(C)** Volcano diagram of DEPs analysis. **(D)** Enriched GO terms of the identified DEPs. **(E)** Enriched KEGG pathways of the DEPs. The top 20 items from GO and KEGG enrichment analysis were displayed. The color gradient indicates the significance of enrichment, and the darker the color, the higher the degree of enrichment (*P value <0.05, **P value <0.01, ***P value <0.001). *P* value by Student’s t-test (two-tailed).

To further clarify the DEPs between HCC and HCC_*Cs* patient serum, totally 16 serum samples of patients (8 cases in each group) were conducted proteomic analyses. The Venn diagram revealed that the HCC group and the HCC_*Cs* group together shared 588 proteins, and individually contained 36 and 59 proteins, respectively (**Figure 4A**). Additionally, clustering analysis results showed that samples within group have high correlation and good sample repeatability (**Figure 4B**). The volcano plot displayed 63 up-regulated DEPs and 43 down-regulated DEPs (FC ≥ 2 or FC ≤ 0.5, *P* < 0.05), and representative up-/down-regulated DEPs were marked in pink ovals and blue ovals, respectively (**Figure 4C**).

The main enriched GO terms included proteasome core complex, intracellular protein-containing complex, proteasome core complex, beta-subunit complex, nucleoplasm, phagocytosis, cellular nitrogen compound biosynthetic process, proteasomal protein catabolic process, proteasome complex, catalytic activity, acting on a nucleic acid, and regulation of inflammatory response to antigenic stimulus. The primarily KEGG pathways involved were proteasome, spinocerebellar ataxia (SCA), Alzheimer disease (AD), Parkinson disease (PD), pathways of neurodegeneration - multiple diseases, amyotrophic lateral sclerosis (ALS), arginine biosynthesis, type I diabetes mellitus, Huntington disease (HD), and Th17 cell differentiation. The DEPs such as PSMB3, PSMB4, PSMB5, PSMA1, PSMA3, ILF2, HSP90 β, PARK7, OLFM4, ASS, and CNN2 were primarily participated in the aforementioned GO terms and KEGG pathways (**Figures 4D, E**).

### 3.5 Clustering and PPI of DEPs in the serum of HCC and HCC_*Cs* patients

**Figure 5:**
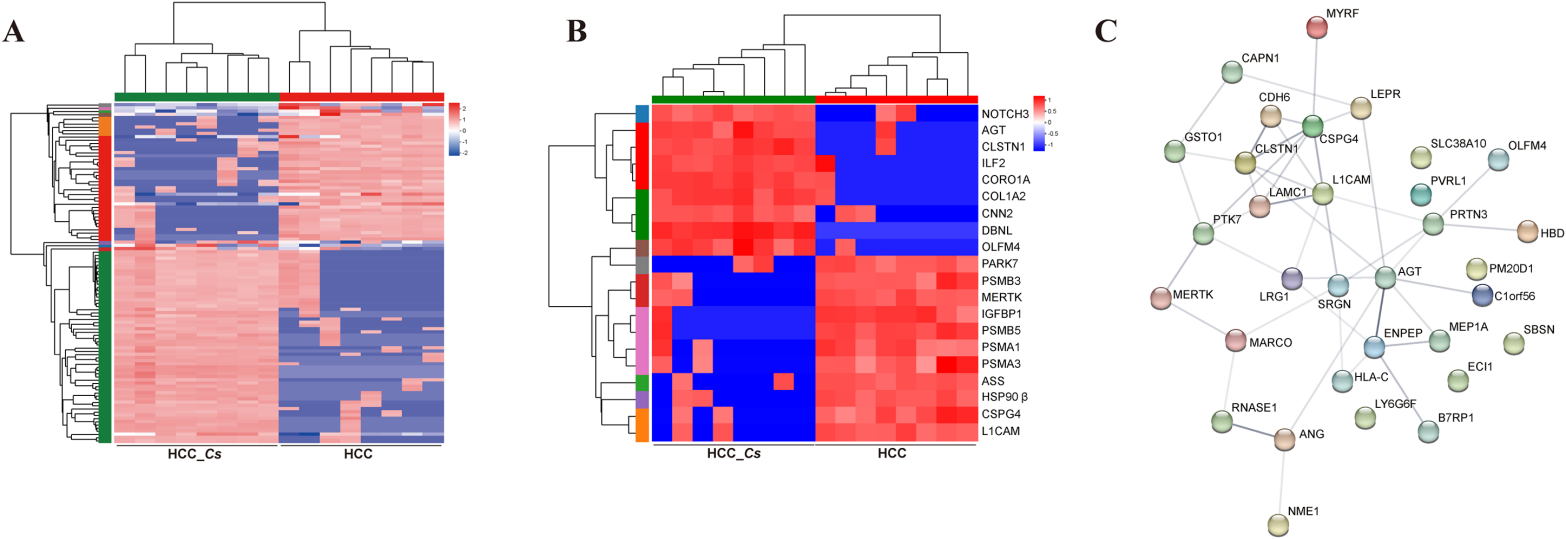
Clustering and PPI network analysis of serum DEPs between HCC group and HCC_*Cs* group. **(A)** Cluster heatmap of the DEPs. **(B)** Cluster analysis of 20 important DEPs. **(C)** PPI network analysis of DEPs.

The results of the expression pattern cluster analysis confirmed that the expression trends of the DEPs in the samples within groups were relatively similar (**Figure 5A**). 20 important DEPs were further selected, clustered and analyzed their expression, including up-regulated CNN2, CDH6, ILF2, CRN, OLFM4, and DBNL, as well as down-regulated TRF, PARK7, HSP90β, PSMB3, IGFBP1, and ASS, etc. (**Figure 5B**). To explore the interaction among DEPs, the PPI network of DEPs was constructed using the STRING database. The proteins at the center of the network were identified as L1CAM, CLSTN1, CSPG4, and AGT (**Figure 5C**).

### 3.6 Functional enrichment analysis of differentially expressed metabolites (DEMs) in the serum of HCC and HCC_*Cs* patients

**Figure 6:**
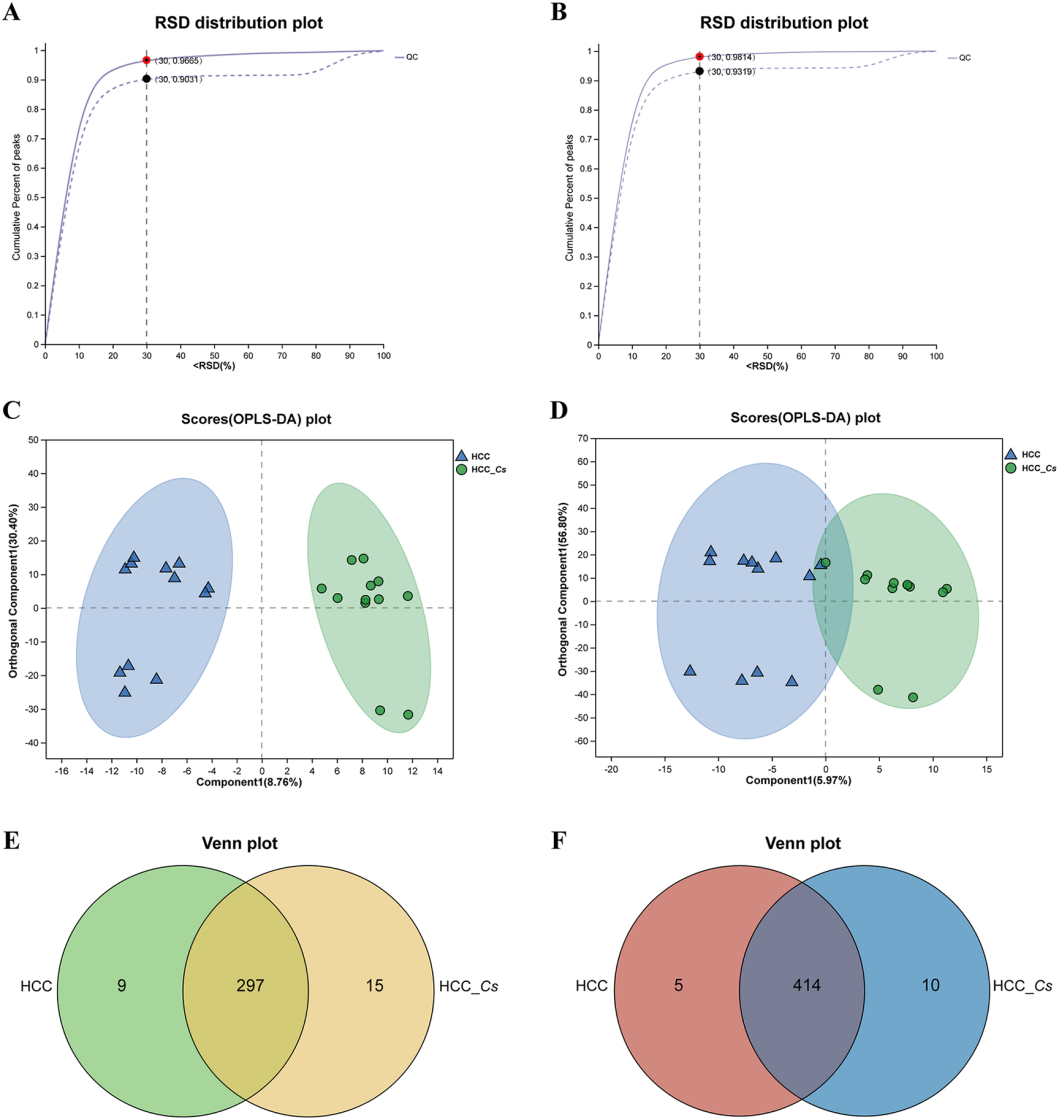
Quality analysis and metabolite identification of serum samples from HCC group and HCC_*Cs* group. RSD evaluation of the reliability of experimental data in negative **(A)** and positive **(B)** ion modes. OPLS-DA analysis of HCC group and HCC_*Cs* group in negative **(C)** and positive **(D)** ion modes. Venn plots displayed the number of metabolites identified between the HCC group and HCC_*Cs* group in negative **(E)** and positive **(F)** ion modes. *P* value by T test/non-parametric test.

**Figure 7:**
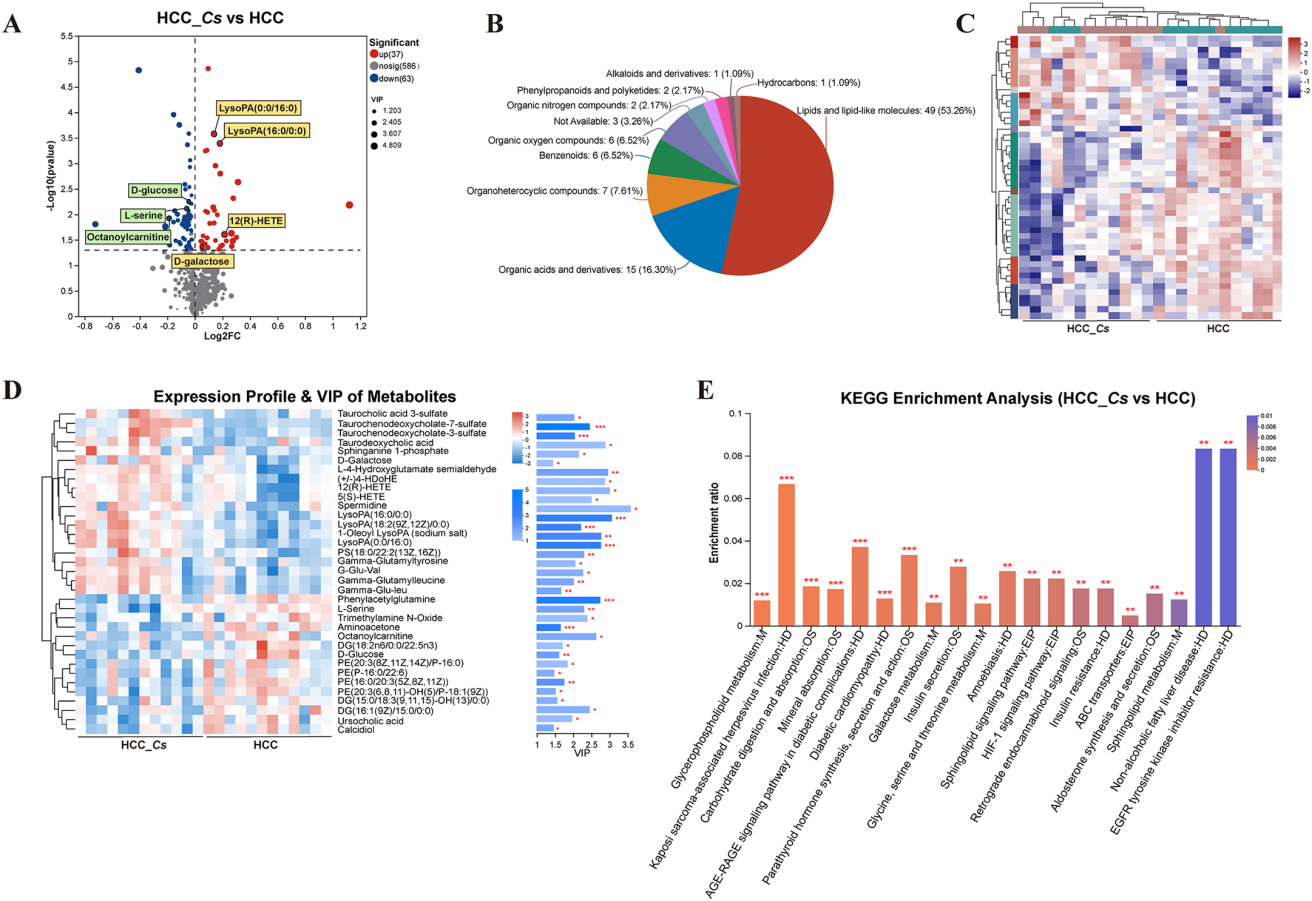
Identification, classification, clustering and enrichment analysis of DEMs. **(A)** Volcano diagram of DEMs. **(B)** Pie chart of classified percentage of DEMs. **(C)** Cluster heatmap analysis of all DEMs. **(D)** VIP scores of the DEMs. **(E)** KEGG enrichment analysis of the DEMs between the HCC group and HCC_*Cs* group. *P <*0.05, *P <*0.01 and *P <*0.001 were marked *, ** and ***, respectively.

To investigate the differences in metabolites between the HCC group and HCC_*Cs* group, untargeted LC-MS/MS was performed on the serum samples from both groups. The reliability of the experimental data was confirmed by the RSD results of the quality control (QC) samples (**Figures 6A, B**). The OPLS-DA model showed small intra-group sample differences and large inter-group sample differences (**Figures 6C, D**). The metabolites detected under the negative and positive ion modes were presented in **Figures 6E, F**, respectively. A total of 100 DEMs were detected, with 37 up-regulated and 63 down-regulated. Representative up- and down- regulated DEMs were marked with yellow rectangles and green rectangles, respectively (**Figure 7A**). The classification diagram of DEMs was shown in **Figure 7B**, and the cluster analysis in **Figure 7C** illustrated the expression changes of the DEMs. Variable importance in projection (VIP) analysis identified the top 35 DEMs that significantly contributed to grouping (VIP ≥ 1, *P* < 0.05). The first 5 metabolites were spermidine, LysoPA(16:0/0:0), 12(R)-HETE, L-4-Hydroxyglutamate semialdehyde, and taurodeoxycholic acid (TDCA) (**Figure 7D**).

KEGG enrichment analysis confirmed that *C. sinensis* infection significantly affected pathways including glycerophospholipid metabolism, carbohydrate digestion and absorption, mineral absorption, AGE-RAGE signaling pathway in diabetic complications, diabetic cardiomyopathy, parathyroid hormone synthesis, secretion and action, galactose metabolism, insulin secretion, glycine, serine and threonine metabolism, and HIF-1 signaling pathway. The DEMs involved in these KEGG enrichment pathways were L-serine, PE(20:3(8Z,11Z,14Z)/P-16:0), DG(15:0/18:3(6Z,9Z,12Z)/0:0), D-galactose, D-glucose, and aminoacetone (**Figure 7E**).

### 3.7 Correlation analysis of serum proteomics and metabolomics of HCC and HCC_*Cs* patients

**Figure 8:**
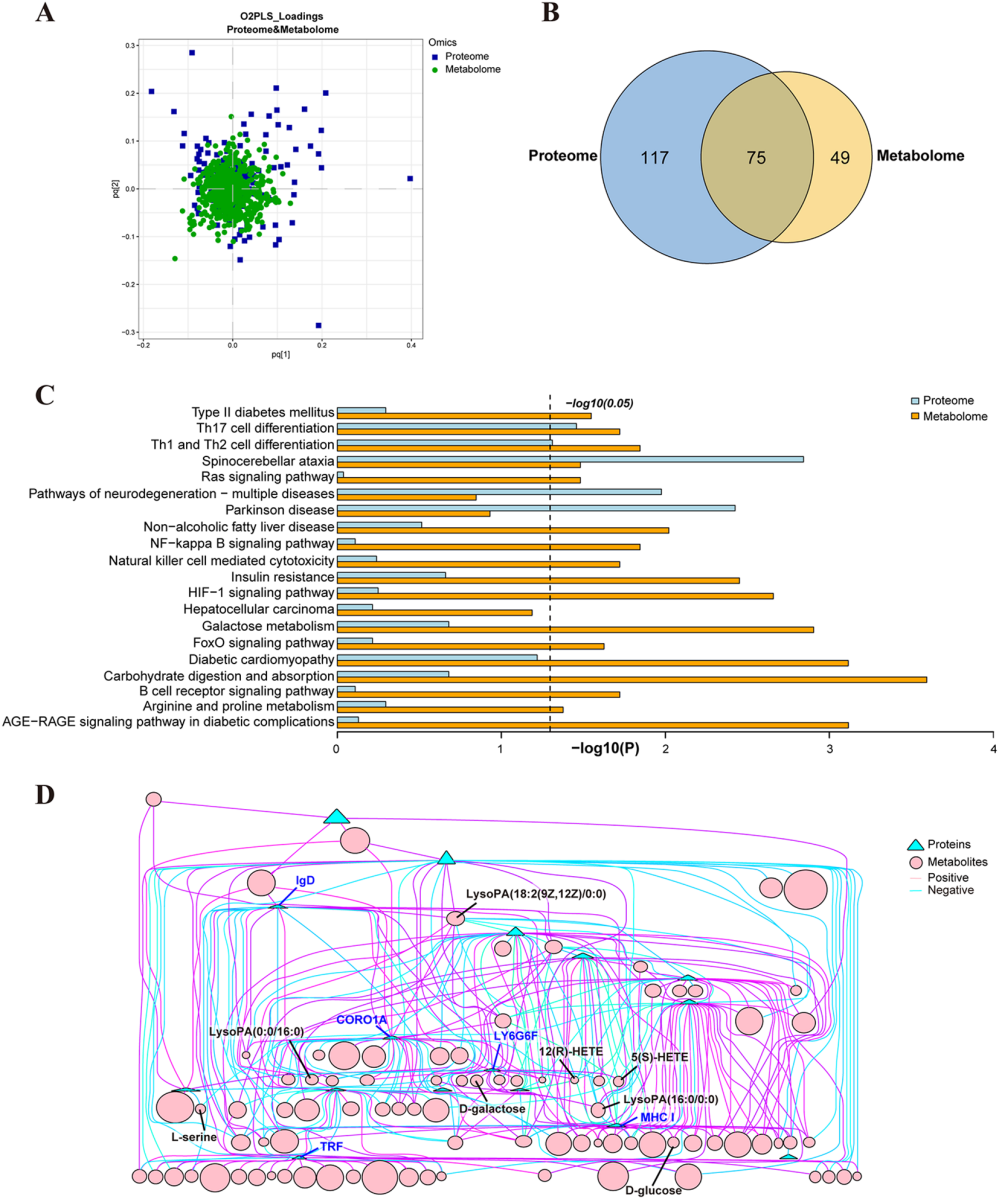
Correlation analysis of serum proteomics and metabolomics from HCC group and HCC_*Cs* group. **(A)** O2PLS analysis of proteomics and metabolomics. **(B)** Venn diagram of KEGG enriched pathways in proteomics and metabolomics. **(C)** 20 co-enriched pathways of proteomics and metabolomics. **(D)** Network diagram of the correlation between DEPs and DEMs.

The O2PLS method was utilized to evaluate the intrinsic correlation between the two datasets. These findings showed a high degree of overlap between the two groups of substances, indicating a strong similarity between them (**Figure 8A**). Functional enrichment analysis and Venn analysis identified a total of 75 pathways co-enriched by proteomics and metabolomics (**Figure 8B**). Moreover, **Figure 8C** displayed 20 crucial co-enriched pathways, encompassing immune and inflammation-related pathways such as Th17 cell differentiation, Th1 and Th2 cell differentiation, and NF-κB signaling pathway, cancer-related pathways like HCC and Ras signaling pathway, neurological diseases related pathways including SCA, PD, and pathways of neurodegeneration - multiple diseases, and diabetes and cardiovascular disease-related pathways such as insulin resistance (IR), type II diabetes mellitus, and diabetic cardiomyopathy. Metabolic pathways closely associated with HCC progression, such as galactose metabolism, carbohydrate digestion and absorption, sphingolipid metabolism, and arginine and proline metabolism, were also co-enriched (**Figure 8C**). The correlation analysis results of DEPs and DEMs unveiled that proteins such as CRN, TRF, MHC I, and IgD formed a complex mutual regulatory network with metabolites such as LysoPA(16:0/0:0), LysoPA(0:0/16:0), 12(R)-HETE, D-glucose, D-galactose, and L-serine (**Figure 8D**). The detailed data of the network list of DEPs and DEMs can be found in **Supplementary Table S1**.

## 4 Discussion

It has been reported that *C. sinensis* infection induces a worse prognosis after hepatectomy in HCC patients (10). Consistently, our nine-year retrospective clinical analysis further confirmed that *C. sinensis* infection did significantly reduce OS and RFS in HCC patients. Specifically, *C. sinensis* infection could significantly up-regulate the proportion of male HCC patients and promote the occurrence of liver cirrhosis and MVI. In addition, our clinical data demonstrated that *C. sinensis* infection significantly affected serum lipid-related indicators of HCC patients, which was manifested by a significant decrease in the levels of APO-A, A1/B1 and HDL-C, while a significant increase in the level of TG.

Metabolic reprogramming is a hallmark of cancer, including HCC (21). Our biomics data revealed significant changes in metabolic pathways in HCC patients infected with *C. sinensis*. Serological proteomics results showed a notable enrichment of the arginine biosynthesis pathway, accompanied by a significant decrease in argininosuccinate synthase (ASS). Arginine plays a crucial role in cancer growth and various aspects of tumor metabolism (22, 23). In healthy cells, ASS is the rate-limiting enzyme responsible for replenishing the arginine pool. However, in most tumors, including HCC, ASS is downregulated or silenced, leading cancer cells to depend on exogenous arginine for survival (22–24). Furthermore, ASS (–) HCC is often poorly differentiated, highly proliferative, and more malignant (24, 25). Therefore, arginine deprivation could be an effective therapy for *C. sinensis* (+) HCC patients. Our serum metabolomics profiling showcased significant alterations in glycerophospholipid and sphingolipid metabolism in *C. sinensis* (+) HCC patients. Recent research has proved that changes in phospholipids and sphingolipids are linked to the onset and progression of primary liver cancer, with variations in bioactive sphingolipids being a characteristic of derived HCC (26, 27). Furthermore, our metabolomics analysis showed significant enrichment of pathways related carbohydrate digestion and absorption, galactose metabolism, and mineral absorption, all of which were associated with a decrease in D-glucose and an increase in D-galactose levels. To keep up with rapid proliferation, HCC, like other cancers, undergoes a reprogramming of glucose metabolism. HCC predominantly relies on glycolysis for ATP production instead of oxidative phosphorylation. This preference for glycolysis, known as the Warburg effect, results in heightened glucose uptake and lactate fermentation (28, 29). Various studies have demonstrated that a low glucose environment can promote migration, metastasis, and cancer stemness in HCC (30, 31). Our biomics data also highlighted the enrichment of the HIF-1 pathway, which is involved in glycolysis, angiogenesis, metastasis, and invasion in cancer (32, 33). These findings suggested that *C. sinensis* infection can exacerbate the malignancy of HCC through metabolic reprogramming, especially by excessive depletion of arginine and D-glucose.

HCC is closely linked to chronic inflammation and fibrosis. Inflammatory cytokines such as IL-6 and TNF-a, along with downstream targets like NF-κB, can drive inflammation-related HCC. Moreover, Th17 cells, CD8^+^ T cells, and B cells can contribute to the development of HCC (34, 35). Our study revealed an imbalance in Th1 and Th2 cell differentiation, as well as an upregulation of IL-6R promoting Th17 cell differentiation in patients with *C. sinensis* (+) HCC through biomics correlation analysis. Additionally, signaling pathways like NF-κB, B cell receptor, and natural killer cell mediated cytotoxicity were also found to be co-enriched. Importantly, our biomics data, particularly the metabolomics, showed an enrichment of various diabetes-related pathways, including type I/II diabetes mellitus, IR, insulin secretion, diabetic cardiomyopathy, and AGE-RAGE signaling pathway in diabetic complications. Numerous studies have confirmed the bidirectional relationship between liver disease and diabetes: advanced liver disease promotes the onset of diabetes, while diabetes is a risk factor for liver fibrosis progression and HCC development, and may worsen the long-term prognosis of HCC patients (36, 37). There is a vicious cycle between IR and inflammation, and the AGE/RAGE/NF-κB axis acts as the nexus for this metabolic disorder (38). Additionally, IR-related hyperinsulinemia promotes the progression of liver fibrosis and HCC by inducing lipotoxic chronic inflammation and increasing the release of inflammatory cytokines (36). Our aforementioned data indicated that *C. sinensis* can drive a vicious cycle of immune inflammation and IR, thereby aggravating the progression of HCC.

Abundant HCC, tumor immunity, and diabetes-related DEPs and DEMs were detected in the present study. Our proteomics data revealed that compared to HCC, the serum proteins of COL1A2, CORO1A, CNN2, DBNL, CDH6, NOTCH3, and ENPEP were significantly increased in patients with *C. sinensis* (+) HCC. These proteins are closely related to tumor proliferation, migration, invasion and metastasis (39–45). ILF2, a key molecule regulator of HCC cell growth and apoptosis, was significantly upregulated (46). Proteins ANG and PTK7, highly expressed in advanced and metastatic HCC, were also significantly increased (47, 48). Moreover, OLFM4, which is linked to tumor stemness and poor prognosis, was significantly expressed (49). Furthermore, our proteomics data revealed dysregulation of serum IGL and IGH fragments in HCC patients with *C. sinensis* infection, particularly those containing the J chain, which were enriched in intestinal immune network for IgA production pathway. It is well-documented that serum IgA can promote liver fibrosis and HCC, while compromising anti-tumor immunity in the liver (50). Excitingly, both HLA-A and HLA-C, important subtypes of human MHC I molecules crucial for tumor recognition and T-cell-mediated elimination, were significantly down-regulated in our study (51, 52). This suggests that *C. sinensis* infection may induce immune system abnormalities and contribute to tumor immune evasion. Furthermore, our proteomic results revealed a significant reduction in the levels of MERTK (beyond the insulin receptor) and IGFBP1, leading to decreased insulin sensitivity and triggering IR and diabetes (53, 54). Additionally, levels of AGT, a biomarker reflecting tubular injury, were significantly elevated, suggesting that *C. sinensis* infection may increase the risk of diabetic nephropathy (55).

Lysophosphatidic acid (LysoPA) has been identified as a hallmark of HCC, involved in the proliferation, metastasis, invasion, survival, and evasion of apoptosis during tumorigenesis (56, 57). Our metabolomic analysis demonstrated that *C. sinensis* infection led to significant elevations in four types of LysoPA and its derivative, namely LysoPA(16:0/0:0), LysoPA(0:0/16:0), LysoPA(18:2(9Z,12Z)/0:0), and 1-Oleoyl LysoPA. Additionally, we observed a significant increase in the production of TDCA, taurochenodeoxycholate-7-sulfate (TCDCA-7S), TCDCA-3S, and taurocholic acid 3-sulfate (TCA-3S) in the serum of *C. sinensis* (+) HCC patients. Previous researches have confirmed that dysregulated hepatic bile acids can synergistically promote the development of liver cancer (58, 59). Furthermore, we found significant upregulation of metabolites associated with liver fibrosis and HCC, including 12(R)-HETE, 5(S)-HETE, and sphinganine 1-phosphate (SA1P) (60–62). Conversely, the levels of octanoylcarnitine and DG(16:1(9Z)/15:0/0:0) were significantly decreased, consistent with previous reports indicating their downregulation in HCC (63, 64). Octanoylcarnitine serves as a serum biomarker for HCC, with levels gradually declining as the disease progresses (63). Moreover, the upregulated 12(R)-HETE, 5(S)-HETE, and spermidine that we detected are closely associated with the development of diabetes. Previous studies have demonstrated that 12-HETE can reduce insulin secretion and increase cell death in human islet cells (65, 66). Similarly, 5-HETE has been shown to promote inflammation in adipose tissue, contributing to diabetes (66). Additionally, elevated levels of spermidine in islet β cells have been implicated in the pathogenesis of diabetes under inflammatory conditions (67). Furthermore, our metabolomic analysis revealed a significant decrease in calcidiol (also known as 25OHD). Low levels of 25OHD have consistently been associated with insulin-resistant diseases, and its serum levels are inversely correlated with the prevalence of diabetes (68, 69).

Surprisingly, our proteomics KEGG analysis revealed that 6 out of the top 10 signaling pathways were related to neurodegenerative diseases, including SCA, AD, PD, pathways of neurodegeneration - multiple diseases, ALS, and HD. Furthermore, correlation analysis results revealed that DEPs and DEMs co-enriched in KEGG pathways of SCA, neurodegeneration-multiple diseases, and PD. The proteasome pathway ranked first in proteomic KEGG enrichment analysis. The DEPs involved in this pathway included upregulated proteasome subunit beta type 4 (PMSB4, highly similar), down-regulated PSMB3, PSMA1, PSMA3, PSMB5, and PSMB2, all of which were involved in the aforementioned degenerative neurological disease pathways. This indicated that *C. sinensis* infection caused significant proteasome impairment in HCC, resulting in malfunction of proteasomal activity triggering protein aggregation, which is a key process in the development of most neurodegenerative diseases (70, 71). Significantly reduced levels of HSP90β and TPP1 in our study led to an increase in misfolded proteins and the formation of abnormal aggregates, which are the main hallmarks of many diseases, including degenerative diseases and cancer (72, 73). In addition, significant reductions in L1CAM, CSPG4, and PARK7 were observed, which can regulate nerve growth and protect nerves from oxidative damage (74–76). Moreover, studies have confirmed that significant alterations in AGT and MERTK are common genes that promote diabetes and AD (53, 77). Additionally, many DEMs closely related to neurodegenerative diseases were measured, such as the up-regulated LysoPA and its derivative, 12(R)-HETE, 5(S)-HETE, and SA1P, and the down-regulated L-serine, D-glucose, and D-galactose. According to existing literature, abnormal expression of LysoPA is linked to the pathogenesis of neurodegenerative diseases, while plasma levels of 5-HETE and 12-HETE are increased in ALS mouse models and PD patients, respectively (78–81). In addition, SA1P in plasma is considered a potential biomarker for AD (82), L-serine deficiency leads to cognitive impairment in AD (83), and abnormal glucose and lactose metabolism are early hallmarks of AD development (84). Overall, *C. sinensis* infection may exacerbate complications of neurodegenerative diseases in HCC.

## 5 Conclusion

**Figure 9:**
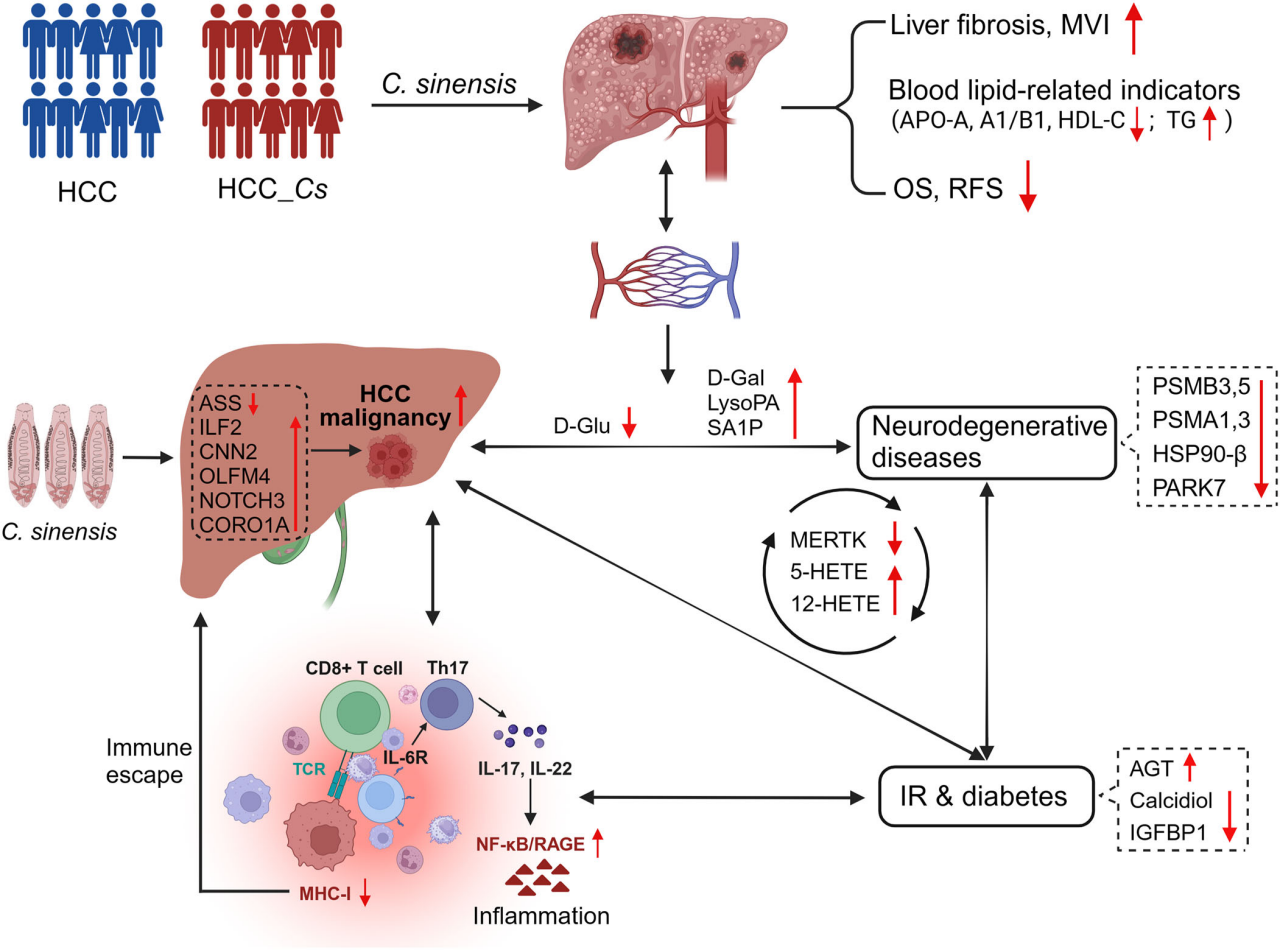
C*. sinensis* infection exacerbated the malignancy of HCC. *C. sinensis* infection was associated with a poor prognosis for HCC, induced metabolic reprogramming in patients with HCC, significantly altered cancer-promoting molecules such as ASS, ILF2, D-Glu and LysoPA, amplified the vicious circle of inflammation and IR, and promoted the occurrence of complications such as degenerative diseases and diabetes.

*C. sinensis* infection could exacerbate liver cirrhosis and MVI, impact blood lipid-related indicators, and significantly reduce OS and RFS in HCC patients. Biomics results validated that *C. sinensis* induced metabolic reprogramming, primarily manifested by the down-regulation of ASS and D-glucose, further exacerbating the malignant progression of HCC. Moreover, *C. sinensis* triggered immune dysregulation in HCC patients, provoking inflammatory reactions, intensifying the detrimental cycle of inflammation and IR, and escalating complications associated with diabetes and neurodegenerative diseases (**Figure 9**).

## Data Availability

The data presented in the study are deposited in the iProX and MetaboLights repository, accession number IPX0008724000 and MTBLS11027, respectively.
