# Supplementary material for: Serum proteomic and metabolomic profiling of hepatocellular carcinoma patients co-infected with Clonorchis sinensis
Source: Front Immunol. 2025 Jan 7;15:1489077. doi: 10.3389/fimmu.2024.1489077 (PMC11746118; doi:10.3389/fimmu.2024.1489077)
Supplement: Supplementary file 1 [file DataSheet1.docx]

Supplementary Material

# Supplementary Figures and Tables

## Supplementary Figures


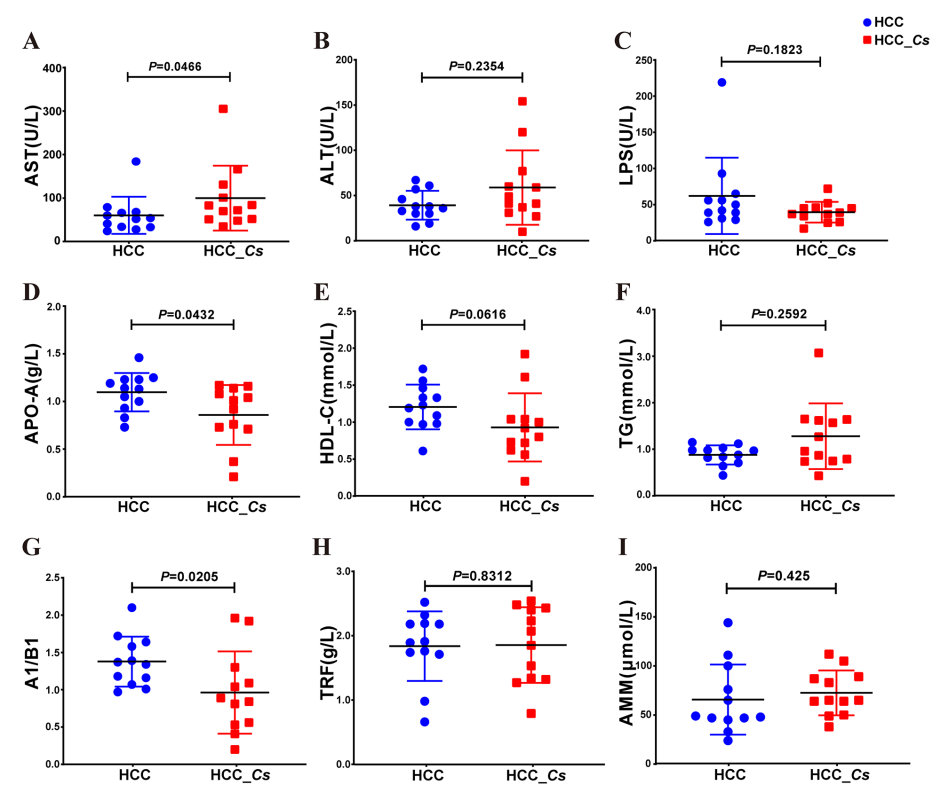


**Supplementary Figure 1.** Serum indicator levels between HCC group and HCC_*Cs* group in small samples. Serum levels of AST (A), ALT (B), LPS (C), APO-A (D), HDL-C (E), TG (F), A1/B1 (G), TRF (H), and AMM (I) in HCC group and HCC_*Cs* group patients were detected. P value by Mann-Whitney U test.

## Supplementary Tables

**Table 1.** Network list of differential proteins and differential metabolites.
